# Integrative ATAC-Seq and RNA-Seq analysis identifies key genes for intramuscular fat content in Laiwu pigs

**DOI:** 10.1186/s12864-026-13135-6

**Published:** 2026-07-02

**Authors:** Jingxuan Li, Jiying Wang, Yanping Wang, Ruihan Mao, Xueyan Zhao

**Affiliations:** 1 Institute of Animal Science and Veterinary Medicine, Shandong Academy of Agricultural Sciences, Jinan, 250100 China; 2 Key Laboratory of Livestock and Poultry Multi-omics of MARA, P.R.China, Jinan, 250100 China; 3 Shandong Provincial Key Laboratory of Livestock and Poultry Breeding, Jinan, 250100 China

**Keywords:** Laiwu pigs, intramuscular fat, ATAC-seq, RNA-seq, transcription factor

## Abstract

**Background:**

Intramuscular fat (IMF) is a key indicator of pork quality. Laiwu pigs represent a valuable model for studying IMF deposition due to their extremely high IMF content, yet the regulatory mechanisms of chromatin accessibility in skeletal muscle remain unclear. To dissect the epigenetic and transcriptional mechanisms underlying IMF deposition, we performed integrative ATAC-seq and RNA-seq analysis on *longissimus thoracis* (LT) muscle of six pigs with extreme IMF phenotypes, selected from a population of 79 Laiwu pigs.

**Results:**

We characterized group-shared accessible chromatin peaks, defined as peaks consistently detected across biological replicates within each group. A total of 22,059 and 14,149 peaks were identified in the high-IMF (IMF-H) and low-IMF (IMF-L) groups, respectively. Motif enrichment analysis of these peaks revealed that IMF-H peaks were enriched for lipid metabolism-related transcription factors (JunB, Fra1, Atf3, Fra2), whereas IMF-L peaks harbored muscle development-related factors (CTCF, Mef2c, Mef2d, BORIS). Comparative analysis between the two groups identified 5,573 upregulated and 3,806 downregulated differential peaks, including 525 group-shared differential peaks. Integrative analysis of ATAC-seq and RNA-seq data uncovered 294 group-shared differential accessible chromatin region (ACR)-linked genes expressed in LT muscle, which were significantly enriched in fat deposition pathways like glycosphingolipid biosynthesis and cholesterol metabolism. Of these genes, 108 were significantly correlated with IMF content in the 79-pig population. Among them, we prioritized promoter ACR-linked *CTSL* (top 10 highest IMF correlation) and *GTF3C3* (with the most significant ACR at its transcription start site), both carrying IMF-associated SNPs in ACRs, as candidate regulators for their potential role in transcriptional control.

**Conclusions:**

This study reveals distinct chromatin accessibility patterns linked to IMF deposition in Laiwu pigs and refines the regulatory network of porcine IMF. The identified candidate genes and regulatory elements provide valuable molecular targets for breeding strategies to improve IMF content and meat quality in pigs.

**Supplementary Information:**

The online version contains supplementary material available at https://doi.org/10.1186/s12864-026-13135-6.

## Introduction

Intramuscular fat (IMF) content is a pivotal trait influencing meat quality, as it directly correlates with tenderness, juiciness, and flavor [1], thereby affecting consumer preference and market value of pork. In recent years, with the continuous growth of global meat demand, meat quality has emerged as a pivotal determinant that increasingly influences consumers’ purchasing preferences and market choices within the pig industry. However, Duroc × (Landrace × Yorkshire) (DLY) hybrid pigs, the most widely consumed commercial pigs and the primary source of pork for consumers, typically have an IMF content of approximately 1% [2]. This level falls below the optimal range of 2.2–3.4%, which is recognized as essential for achieving superior meat palatability [3]. Meanwhile, improving IMF content through traditional breeding approaches remains considerably challenging, largely owing to the prolonged duration, high costs, and reliance on post-slaughter assessment for IMF content. Consequently, deciphering the genetic regulatory mechanisms underlying IMF deposition is pivotal for facilitating the improvement of IMF content via molecular breeding strategies.

Skeletal muscle serves as the primary site for IMF deposition, where IMF exists both as triglycerides within myocytes (particularly abundant in oxidative myofibers) and as lipid droplets in intramuscular adipocytes [4, 5]. These adipocytes, derived from the differentiation of intramuscular preadipocytes, accumulate between myofibers or around muscle bundles, forming intramuscular adipose tissue [6]. The process is tightly regulated by dynamic changes in gene expression, which are in turn modulated by epigenetic mechanisms, such as chromatin accessibility. Chromatin accessibility refers to the degree of physical contact between nuclear macromolecules and chromatinized DNA, whose level is tightly regulated by the topological organization of nucleosomes, nucleosome occupancy, and the binding of chromatin-interacting factors [7]. Assay for Transposase-Accessible Chromatin using sequencing (ATAC-seq) is a quantitative method to measure sites-specific chromatin accessibility using Tn5 transposase to insert adaptors into accessible chromatin loci, thereby providing insights into potential regulatory elements that drive cell-type-specific gene expression [8–10]. Notably, the accessible genome makes up approximately 2–3% of the total DNA sequence, yet it encompasses over 90% of the regions bound by transcription factors (TFs) [11]. When combined with RNA sequencing (RNA-seq), which could capture the transcriptional landscape and identify dynamically expressed genes during biological processes, ATAC-seq enables the construction of an integrated understanding of gene expression regulatory networks. Specifically, genes whose promoter regions exhibit chromatin accessibility tend to show differential expression at the mRNA level, and this expression is likely to be regulated by TFs [12].

Since its first application in 2013, ATAC-seq has widely used in conjunction with RNA-seq, contributing to research on human diseases [13, 14] and complex traits in animal species [15–17]. In farm animals, the integrative analysis of ATAC-seq and RNA-seq has been employed to develop a comprehensive understanding of how chromatin accessibility dynamics regulate gene expression patterns associated with traits associated with meat quality such as muscle development [18–21], IMF content [22, 23], adipocyte differentiation [24]. For IMF content trait in pigs, studies were carried out in a composite breed between groups with extreme IMF contents [22], and in porcine intramuscular preadipocytes during its adipogenic differentiation [23].

Among Chinese indigenous pig breeds, Laiwu pigs are renowned for their exceptionally high IMF content (often exceeding 10%) [25], making them an invaluable model for unraveling the genetic and molecular mechanisms underlying IMF deposition. To date, single-omics or muti-omics have been conducted in Laiwu pigs to identify critical single nucleotide polymorphisms (SNPs), genes, non-coding RNAs, metabolites, and microbes associated with IMF content [26–31]. However, progress in identifying breed-specific regulatory elements and critical genes in this breed remains limited. More notably, the regulatory networks governing IMF accumulation in Laiwu pigs, particularly the interplay between chromatin accessibility and gene expression in skeletal muscle tissues, remain poorly understood.

Since IMF deposition is tightly regulated by the crosstalk between muscle cells and adipocytes within the *longissimus thoracis* (LT) muscle [32], studying the LT muscle offers a direct window to dissect the reciprocal regulatory mechanisms underlying IMF accumulation. Given the unique genetic background and extreme IMF phenotype of Laiwu pigs, integrating ATAC-seq and RNA-seq to analyze the LT muscle could uncover novel regulatory nodes, including critical genes and motifs, that specifically contribute to clarifying the biological basis of IMF deposition. Thus, the present study aims to integrate ATAC-seq and RNA-seq data from LT muscle of Laiwu pigs to identify critical genes and regulatory elements associated with IMF content. By linking open chromatin regions to their corresponding expressed genes, we seek to unravel the regulatory networks governing IMF deposition, ultimately contributing to deepen our understanding of meat quality traits in Chinese indigenous pig breeds and provide support for improving meat quality in pig breeding programs.

## Materials

### Animals and Sample Collection

A total of 79 Laiwu pigs used in this study were obtained from the Laiwu Pig Breeding Farm (Jinan, China). Pigs were fed formulated diets with consistent nutrient content and had access to water according to their age. The pigs were slaughtered at the same slaughterhouse after being stunned with an electric shock when they reached approximately 100 kg in weight. Around 200 g of LT tissue from the last fourth and fifth thoracic vertebrae was collected for IMF content determination via the Soxhlet petroleum-ether method (as described previously [33]). A subset of this tissue was immediately preserved in liquid nitrogen for ATAC-seq and RNA-seq analyses. From the LT tissue, we prepared a 1 cm × 1 cm × 1 cm muscle block, ensuring its orientation was parallel or perpendicular to the muscle fibers. These muscle blocks were then submerged in a 4% paraformaldehyde solution for subsequent Oil Red O staining.

### Oil red O staining

The lipid content of muscle fibers of individuals in the IMF-H and IMF-L groups was measured using the oil red O staining technique. Briefly, the oil red O working solution was prepared at 3:2 ratio of saturated oil red O dye solution (Servicebio, China) to distilled water, followed by filtration through qualitative filter paper for clarification. Prior to staining, muscle blocks were fixed in paraformaldehyde solution for over 12 h, then embedded in OCT compound and frozen to achieve the desired hardness. Cryostat sectioning was performed at -20 °C, and the resulting tissue slices were carefully placed onto glass slides. Subsequently, the slices were immersed in the oil red O working solution for 8–10 min to achieve effective lipid staining, followed by differentiation with 60% isopropyl alcohol. For nuclear staining, the sections were briefly dipped in hematoxylin for 3–5 min, then rinsed three times with pure water to eliminate residual staining solutions. Finally, the slides were overlayed with a glycerol drop and inspected under a microscope.

### ATAC-seq and data analysis

Six samples were selected based on the IMF content and sex to construct libraries for ATAC-seq analysis. The ATAC-seq procedure was performed following a previously reported method [34]. Briefly, frozen tissues were ground in the cold homogenization buffer, and nucleus suspensions were obtained following density gradient centrifugation for 10 min. Nuclei extracted from approximately 50,000 cells were subjected to transposition by resuspension in Tn5 transposase reaction mix (Nextera, USA) and incubating at 37 °C for 30 min. After transposition, equimolar amounts of Adapter 1 and Adapter 2 were added to the mixture, followed by PCR amplification; libraries were then purified using AMPure beads (Beckman, USA). Library quality was assessed with Qubit Fluorometer (Thermo, USA), and index-coded samples were clustered on a cBot Cluster Generation System using TruSeq PE Cluster Kit v3-cBot-HS (Illumina, USA) in accordance with the manufacturer’s instructions. Finally, the prepared libraries were sequenced on an Illumina NovaSeq platform, generating 150 bp paired-end reads.

For all samples, raw data in FASTQ format were processed using fastp (v0.20.0) to generate clean data by removing reads containing adapter, ploy-N and low-quality reads. Clean reads were aligned to pig reference genome (*Sus scrofa* 11.1) using Burrows-Wheeler Aligner (BWA) with default parameters. Reads mapped to mitochondrial DNA were discarded, and only high-quality reads (Mapping Quality ≥ 13) and properly paired reads (length > 18 nt) were retained for subsequent analysis. DeepTools was used to characterize read distribution at and around the transcriptional start sites (TSS) and across gene bodies, while the Integrative Genomics Viewer (IGV) were used to visualize the genome-wide read coverage using BAM alignment files. Peak calling was performed with MACS2 (v2.1.0) using the parameters: macs2 callpeak -q 0.05 -f AUTO --call-summits --nomodel --shift − 100 --extsize 200 --keep-dup all. Peaks with a fold change (FC) in reads per million (RPM) ≥ 1.5 were defined as differential peaks. Furthermore, to improve the reliability and robustness of screening results, we combined the FC threshold criterion with within-group replicate-consistent filtering to obtain high-confidence differential ACRs for subsequent analysis. ChIPseeker was used to retrieve the nearest genes to each peak and annotate the genomic regions of the peaks. Motif discoveries were performed using findMotifsGenome.pl program in HOMER v4.9.1.

### RNA sequencing and whole-genome resequencing

In our previous study, we performed transcriptome sequencing and whole-genome resequencing on a total of 79 Laiwu pigs [33]. Briefly, LT muscle samples were subjected to RNA-seq using the Illumina NovaSeq platform, and ear tissue samples were used for whole-genome resequencing on the DNBSEQ-T7 platform with an average sequencing depth of 10.85×. All raw sequencing data from the above datasets have been deposited in public databases with accessible accession numbers (GSA: CRA019689, CRA019814) (https://ngdc.cncb.ac.cn/gsa). Additionally, a correlation analysis between gene expression profiles and IMF content was also performed in that study [33]. For the present study, association analysis was newly performed using the “-assoc” module in PLINK, with SNPs from the whole-genome resequencing data and corresponding IMF content values variables. Notably, although the original sequencing data were generated and partially analyzed in our previous report, all multi-omics integration strategies, open chromatin region identification, combined omics analysis and candidate gene identification conducted in this manuscript are independently designed and newly completed, which ensures the independence and novelty of the present research.

### Validation of gene expression and genotypes

To validate the reliability of the RNA-seq data, 13 samples were randomly selected from the 79 Laiwu pigs for quantitative real-time PCR (qRT-PCR) analysis. Total RNA was isolated using RNAIso Plus reagent (Takara, Dalian, China) and then reverse transcribed into cDNA with PimeScript RT reagent Kit with gDNA Ereaser (Takara), following the manufacturer’s instructions. qRT-PCR was performed using TB Green Premix Ex Taq II (Takara) on an ABI QuantStudio 5 system (Applied Biosystems, USA). The primers of *CTSL* and *GTF3C3* used were designed by Primer 5.0 software and are listed in Table 1. Thermal cycling conditions were as follows: initial denaturation at 95℃ for 30 min, followed by 40 cycles of 95℃ for 5 s and 60℃ for 30 s, and with melt curve stage at 95℃ for 15 s, 60℃ for 1 min and 95℃ for 1 s. Gene expression levels were normalized to the reference gene *TBP*, and each assay was performed in three biological replicates.

**Table 1:** Primers used for qRT-PCR and PCR validation

Name	Purpose	Primer sequence (5’ to 3’)
GTSL-RT-FP	qRT-PCR	GAAGCACAAGAAGGGAAAAG
GTSL-RT-RP	qRT-PCR	AAAGCCCAACAAGAACCACA
GTF3C3-RT-FP	qRT-PCR	GAGTTACTTGTGGATTCTTC
GTF3C3-RT-RP	qRT-PCR	CCTGATATAGTTGTATGCCT
TBP-RT-FP	qRT-PCR	CAGAACAATAGCCTTCCACCT
TBP-RT-RP	qRT-PCR	GGAGTCAGTCCTGTGCCATAA
GTSL-FP	PCR	GGTCACTGTCCCAACTTCA
GTSL-RP	PCR	ACTCAGAGCCTGTTCCCGC
GTF3C3-FP	PCR	AGTAACCCCATTCGCTCTAA
GTF3C3-RP	PCR	CCAGGTAAACCAGGTCATCA

To validate key SNPs located within the ACR of the *CTSL* and *GTF3C3* genes, three individuals representing each genotype of the target SNPs were randomly selected. Genomic DNA was extracted using TIANamp Genomic DNA kit (TIANGEN, Beijing, China). Target DNA fragments harboring the SNPs were amplified by PCR using 2×Taq Master Mix (Vazyme, Nanjing, China), with the following cycling conditions: initial denaturation at 95 °C for 5 min, 35 cycles of 95 °C for 15 s, annealing at 50 °C for *CTSL* and 60 °C for *GTF3C3* for 30 s, and 72 °C for 1 min, followed by a final extension at 72 °C for 5 min. Sanger sequencing of the PCR products was performed by Tsingke Biotechnology Co., Ltd (Beijing, China).

## Results

### Phenotype of IMF-H and IMF-L groups in Laiwu pigs

**Fig. 1:**
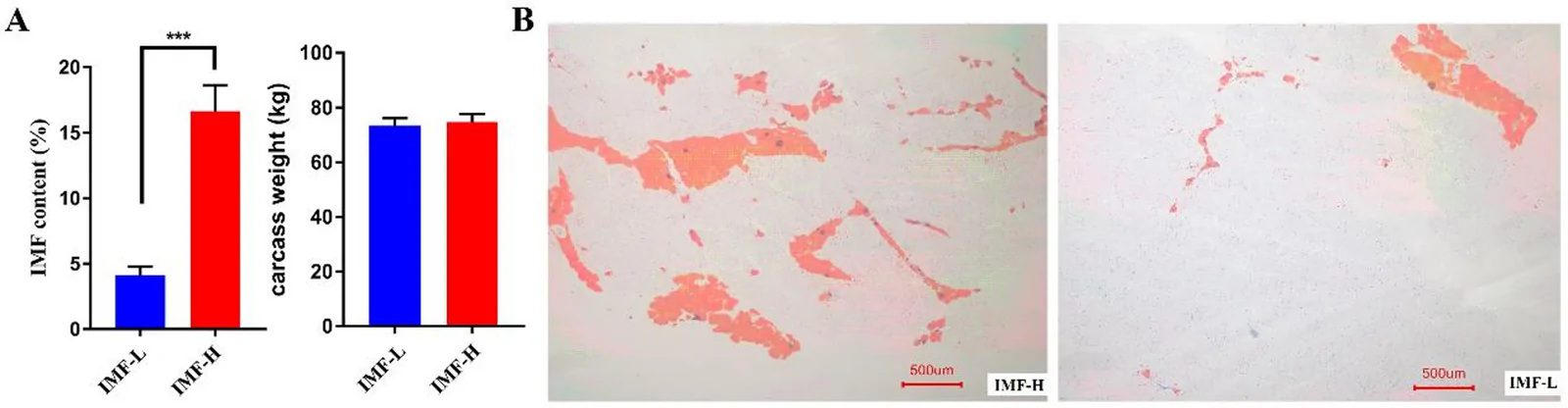
Phenotype of high intramuscular fat (IMF) content (IMF-H) and low IMF content (IMF-L) groups. **A** IMF content and carcass weight in the IMF-H and IMF-L groups. ***, *p* < 0.001. **B** Oil red O staining of *longissimus thoracis* from IMF-H and IMF-L pigs

**Fig. 2:**
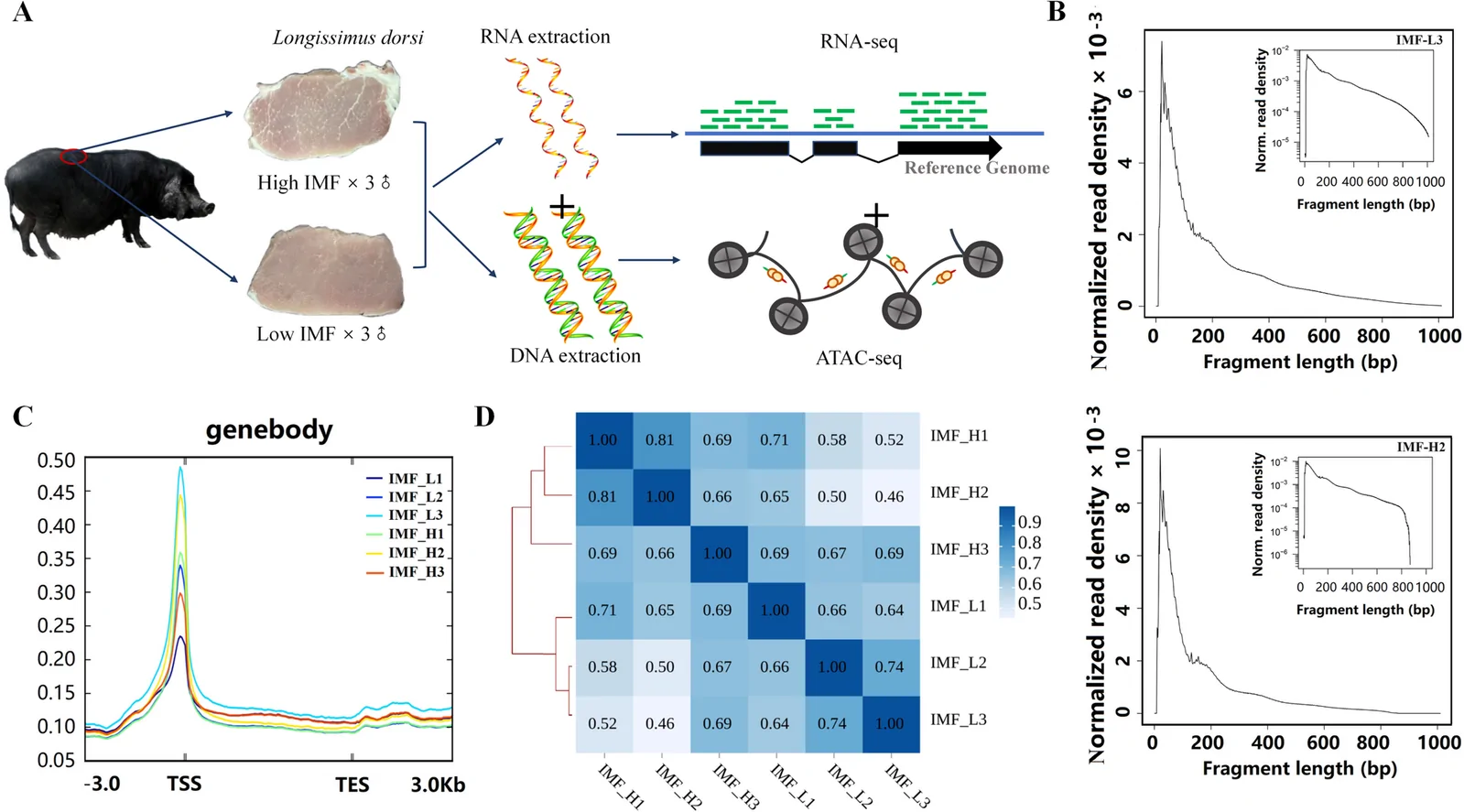
Overview of the ATAC-seq libraries. **A** Schematic diagram of the experimental workflow. IMF, intramuscular fat. **B** Fragment length distribution profile of a representative individual from high IMF (IMF-H) and low IMF (IMF-L) groups. **C** Enrichment of ATAC-seq signals around the transcription start site (TSS). TES, transcription end site. **D** The Pearson’s correlation based on genome‑wide accessible chromatin signals shown as a heatmap scatterplot

To select samples for ATAC-seq and RNA-seq, the IMF content of LT muscle was determined in 79 Laiwu pigs. The IMF content exhibited substantial individual variation, ranging from 3.19% to 18.57%. Based on the IMF content and sex, six boars with extremely high or low IMF content were selected and assigned to two groups: a high IMF content (IMF-H) group and a low IMF content (IMF-L) group. The IMF content of the two groups was 16.64 ± 1.98% and 4.13 ± 0.64%, respectively, showing a significantly difference (*p* < 0.001, Fig. 1A). However, the two groups had a similar average carcass weight of approximately 74 kg, with no significant difference (Fig. 1A). Oil Red O staining of LT muscle sections was consistent with the determination of IMF content. Compared with the IMF-L group, significant more oil red O-stained lipid droplets were observed in the LT muscle sections of the IMF-H group (Fig. 1B). All six samples were subjected to ATAC-seq analysis, and a schematic overview of the study design and analytical pipeline is presented in Fig. 2A.

### ATAC-seq quality control of the LT muscle

To investigate the genome-wide accessible chromatin regions involved in IMF deposition, we profiled the accessibility of chromatin between the IMF-H and IMF-L groups of Laiwu pigs using ATAC-seq. A total of 283,756,415 raw reads were obtained, with 241,458,574 clean reads uniquely mapped to the reference genome after quality filtration. Sequencing and mapping statistics for the reference genome-aligned reads are presented in Table 2. Libraries quality was evaluated based on the insert lengths and peak signal distributions. Fragment size distribution exhibited a multi-modal profile (Fig. 2B): a prominent peak at short fragment lengths, consistent with nucleosome-free open chromatin, was accompanied by smaller peaks around 200 bp and 400 bp—corresponding to mono- and dinucleosome-associated fragments, respectively—consistent with the characteristic pattern of high-quality ATAC-seq data. Further, most identified peaks were across the gene bodies and significantly enriching in the 3 kb of TSS—a hallmark feature of functionally active open chromatin involved in transcriptional regulation (Fig. 2C). Taken together, these quality control analyses demonstrate that our ATAC-seq data are reliable and of high quality for subsequent analyses. Furthermore, sample correlation heatmap based on genome‑wide accessible chromatin signals confirmed high intra‑group consistency and clear separation between the IMF‑H and IMF‑L groups (Fig. 2D), further validating the robustness of our biological replicates.

**Table 2:** ATAC-seq and reference genome mapping statistics of six Laiwu pigs with extremely high or low intramuscular fat (IMF) content

Sample	IMF-H1	IMF-H2	IMF-H3	IMF-L1	IMF-L2	IMF-L3
Raw reads	51,785,399	45,538,869	47,133,093	45,981,335	45,631,149	47,686,570
Raw bases (G)	15.54	13.66	14.14	13.79	13.69	14.31
Clean reads	50,610,208	44,192,242	45,312,458	44,108,608	43,506,312	45,798,154
Clean bases (G)	9.13	8.72	10.25	9.69	8.88	9.73
Clean ratio	58.75%	63.84%	72.49%	70.27%	64.86%	67.99%
Unique mapped	43,529,698	39,657,322	40,990,486	39,146,918	36,998,560	41,135,590
Unique mapped ratio	92.92%	94.50%	95.30%	94.60%	93.95%	95.22%

IMF-H: Individuals with high IMF content

IMF-L: Individuals with low IMF content

Clean ratio: Percent of clean reads to the total number of raw reads

Unique mapped ratio: Number of reads with a unique mapped position on the reference sequence

### Genome-wide identification of accessible chromatin regions (ACRs) in IMF-H and IMF-L groups

**Fig. 3:**
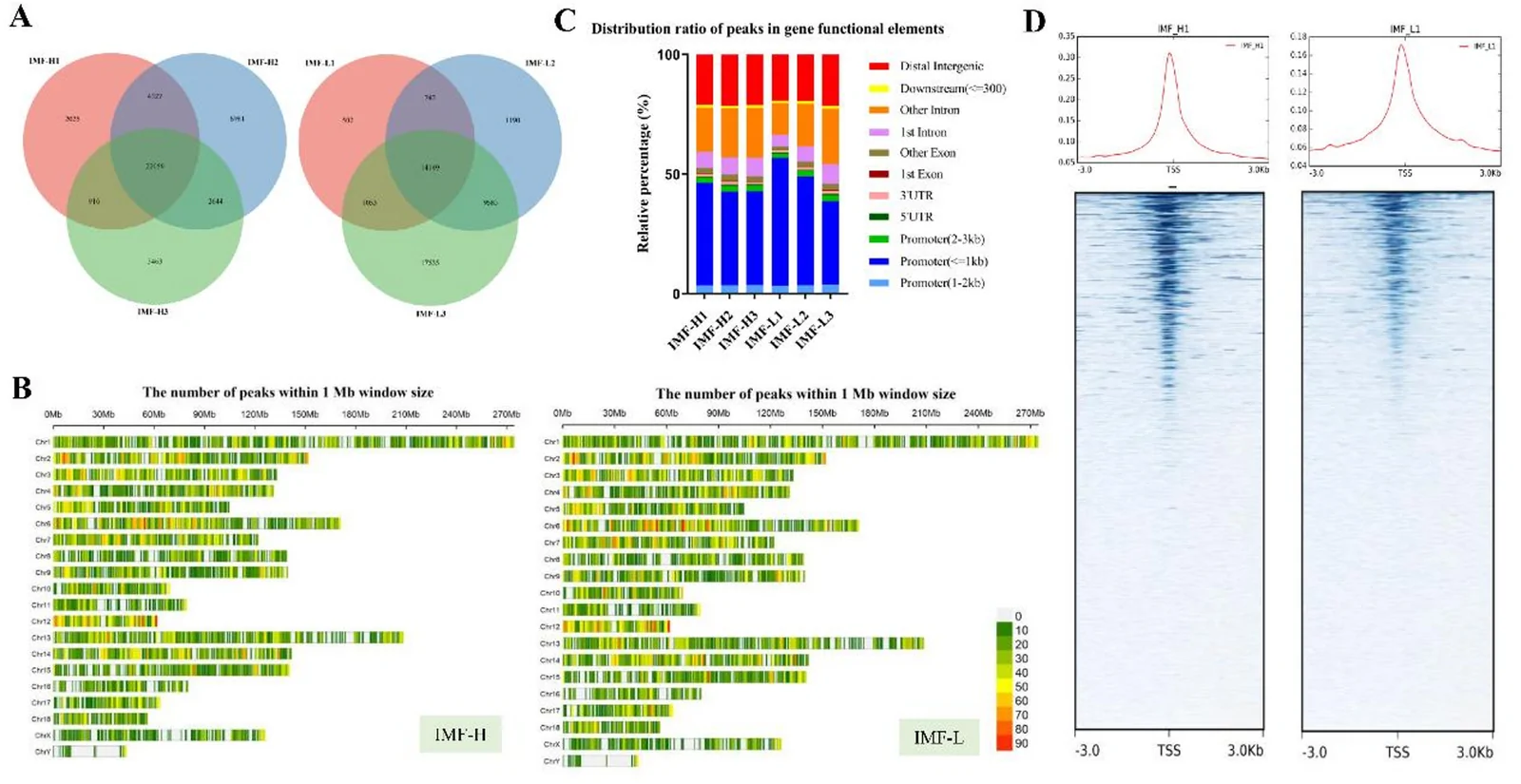
Characterization of accessible chromatin peaks in high intramuscular fat (IMF) content (IMF-H) and low IMF content (IMF-L) groups. **A** Venn diagram of peaks in IMF-H and IMF-L groups. **B** Chromosomal distribution of peaks across the pig genome in IMF-H and IMF-L groups. **C** Distribution of peaks across gene functional elements. **D** Heatmap of peaks within ± 3 kb of gene transcription start site (TSS)

We identified 42,415 and 44,254 accessible chromatin peaks in IMF-H and IMF-L groups, respectively. The peaks consistently detected across biological replicates within each group were defined as group-shared peaks. A total of 22,059 such peaks were obtained in the IMF-H group and 14,149 in the IMF-L group (Fig. 3A). Chromosomal distribution of the peaks across the pig genome is shown in Fig. 3B: most regions across all chromosomes were covered, though sparse coverage was observed for specific chromosomes (e.g., Chr X and Chr Y). To further profile the ACRs distribution across the whole genome, we evaluated the relative genomic positions of the peaks. No significant between-group difference in ACR distribution was detected (Supplementary Table 1). As shown in Fig. 3C, for all samples, most peaks mapped to promoter (48.39% ± 2.26%), intronic (25.85% ± 1.69%), and intergenic regions (20.62% ± 0.35%), and these accounted for approximately 95% of total peaks. Compared with intronic region, few peaks were identified in exonic regions (2.50% ± 0.17%). Among the peaks located in promoter regions, about 87.45% were annotated within 1 kb of the TSS; approximately 26.16% of intronic peaks were annotated in the first intronic. Heatmap analysis of peaks within ± 3 kb of gene TSSs revealed prominent signal enrichment near TSSs, indicating high chromatin accessibility in this region across both groups (Fig. 3D). Motif enrichment analysis revealed that the top 10 significantly enriched TF binding motifs among the group-shared peaks in IMF-H group included binding sites for JunB, Fra1, Atf3 and Fra2—all of which are well-documented regulators associated with adipocyte differentiation and lipid metabolism (Fig. S1A). In contrast, the top enriched motifs in IMF-L group were dominated by binding sites for CTCF, Mef2c, Mef2d and BORIS, which are key factors involved in skeletal muscle development and myogenesis (Fig. S1B). Consistent with the motif enrichment results, transcriptome-based expression profiling of these transcription factors revealed clear group-specific patterns (Fig. S1C). Hierarchical clustering of the samples based on TF expression levels clearly distinguished the IMF-H and IMF-L groups.

### Differential analysis reveals altered chromatin accessibility between IMF-H and IMF-L groups

**Fig. 4:**
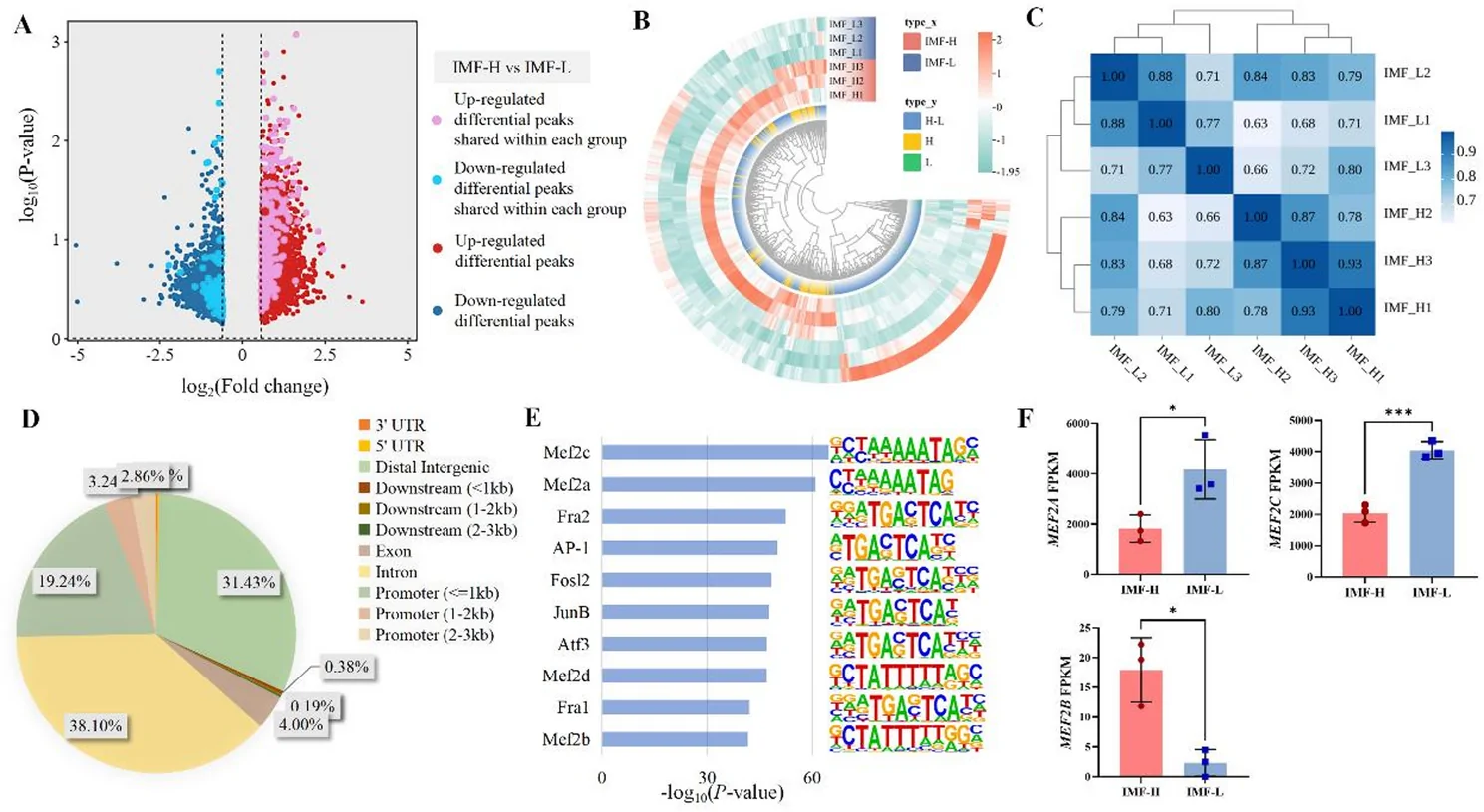
Differentially accessible chromatin peaks between high intramuscular fat (IMF) content (IMF-H) and low IMF content (IMF-L) groups. **A** Scatter plot of differential peaks and group-shared differential peaks. **B** Heatmaps of group-shared differential peaks. H-L: peaks distributed across all six individuals; H: peaks specifically distributed in the IMF-H group; L: peaks specifically distributed in the IMF-L group. **C** The Pearson’s correlation based on 525 group-share differential accessible chromatin regions shown as a heatmap scatterplot. **D** Distribution of group-shared differential peaks across gene functional elements. **E** Top 10 significantly enriched transcription factor binding motifs of group-shared differential peaks. **F **The expression levels of *MEF2A*, *MEF2C*, and *MEF2B* in IMF-H and IMF-L groups. *, *p* < 0.05; ***, *p* < 0.001

To better visualize the loci with altered chromatin accessibility in response to IMF content, we compared the ATAC-seq peaks identified in the IMF-H and IMF-L groups in the term of FC below 1.5. There were 5,573 upregulated (more accessible chromatin) and 3,806 downregulated (less accessible chromatin) peaks in the IMF-H compared with IMF-L group (Fig. 4A). By annotating these different peaks, more accessible chromatin of the IMF-H group corresponded to 4,050 genes, whereas that of the IMF-L group corresponded to 2,629 genes (Table S2). Considering the inherent cellular heterogeneity of skeletal muscle tissue, we further screened 525 group-shared differential peaks that were reproducible across biological replicates within each group. These peaks were defined as high-confidence differential ACRs for subsequent analysis (Fig. 4A). Among these peaks, 399 exhibited differential distribution across the six individuals, and 126 showed strict group-specific distribution: they were exclusively detected in all three individuals of either the IMF-H group or IMF-L group, implying distinct gene regulatory programs underlying differential IMF deposition (Table S3). Specifically, the IMF-H group showed 359 upregulated and 166 downregulated high-confidence differential peaks relative to the IMF-L group (Fig. 4B).

To further validate the reproducibility of these high-confidence signals, we performed sample correlation analysis based on the 525 differential ACRs was carried out. As shown in Fig. 4C, all samples exhibit high overall correlation (*r* = 0.63–0.93), with biological replicates within each group exhibiting among the highest pairwise similarities (e.g., IMF-H1 vs. IMF-H3: *r* = 0.93; IMF-L1 vs. IMF-L2: *r* = 0.88). These results confirm strong intra-group reproducibility of our filtered differential signals. These differential peaks were predominantly mapped to intron (38.25%), distal intergenic (32.57%), and promoter (23.81%) regions (Fig. 4D). Motif enrichment analysis of these 525 peaks revealed that the top 10 significantly enriched TF binding motifs correspond to the binding sites of Mef2 family members, including Mef2a, Mef2c, Mef2d, and Mef2b (Fig. 4E). In addition, we combined transcriptome data to analyze the expression patterns of these genes. The expression levels of *MEF2A* and *MEF2C* were markedly higher in the low IMF group, while *MEF2B* was significantly upregulated in the high IMF group (Fig. 4F).

### Integration of ATAC-seq results with RNA-seq

**Fig. 5:**
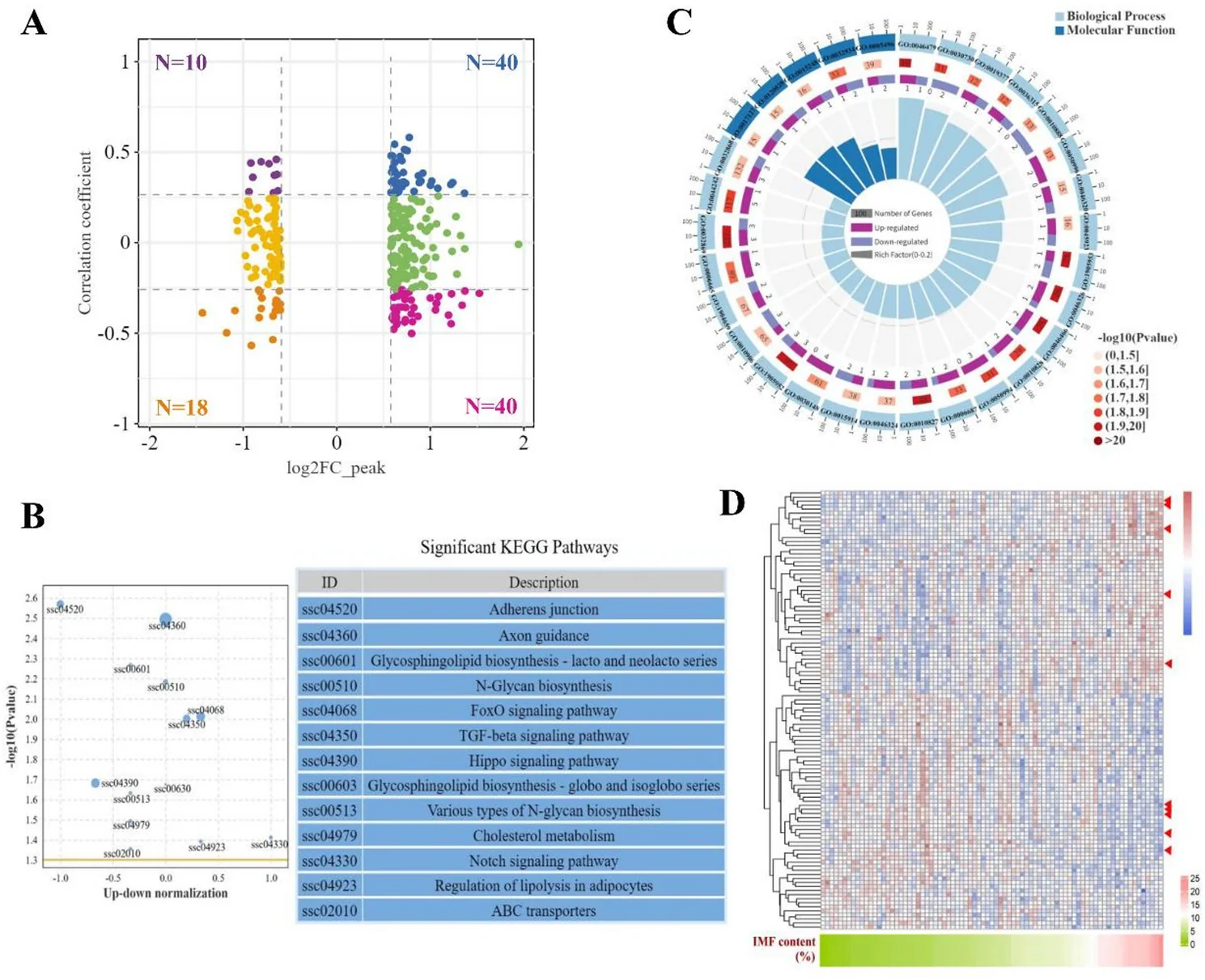
Multi-omics integration of ATAC-seq and RNA-seq data. **A** Nine-quadrant plot of 294 group-shared differential accessible chromatin region (ACR)-linked genes expressed in at least half of the 79 Laiwu pigs. The x-axis represents log_2_ fold change (log_2_FC) of ACRs corresponding to the target genes; the y-axis represents correlation coefficient between log₂-transformed gene expression and correct intramuscular fat (IMF) content. **B** Significantly enriched pathways (*p* < 0.05) from group-shared differential ACR-linked genes. Up-down normalization represents an index for quantifying the imbalance degree of up-regulated and down-regulated genes in the pathways, calculated as (*up* - *down*)/(*up* + *down*) where *up* denotes the number of up-regulated genes and *down* denotes the number of down-regulated genes in the pathway. **C** To 30 significantly enriched Gene Ontology (GO) terms (*p* < 0.05) from group-shared differential ACR-linked genes. **D** Expression heatmap of 108 group-shared differential ACR-linked genes with significant correlation to IMF content across the 79 Laiwu pigs

To further analyze the potential functional implications of the 525 group-shared differential ACRs associated with IMF content, we annotated these ACRs to their corresponding genes and performed pathway enrichment analysis. Our results indicated that these 525 ACRs were linked to 500 genes. Based on previously generated RNA-seq data [33], genes were considered expressed and retained for subsequent analysis if their FPKM > 0.1 in at least 50% of the 79 Laiwu pig individuals. In total, 294 out of these 500 genes met this expression criterion. These 294 expressed genes were linked to the 69 ATAC-seq upregulated peaks and 148 ATAC-seq downregulated peaks; 135 of these genes exhibited RNA-seq upregulation and 159 showed RNA-seq downregulation correlated with IMF content across the 79-individual cohort (Fig. 5A). These 294 genes were significantly enriched in pathways related to glucose metabolism, lipid metabolism, and energy homeostasis, including Glycosphingolipid biosynthesis [35], Cholesterol metabolism [36], FoxO signaling pathway [37, 38], and Hippo Signaling pathway [34] (Fig. 5B, Table S4). Parallel enrichment patterns were also observed among significantly enriched GO terms, such as regulation of lipid localization, positive regulation of glucose import, glycosphingolipid catabolic process, sequestering of triglyceride, and sterol binding (Fig. 5C, Table S4).

In our previous study, a linear model was used to correct IMF content and log₂-transformed gene expression data in 79 Laiwu pigs, with sex and slaughter batch as fixed effects and carcass weight as a covariate [33]. Pearson correlation analysis was then performed between residuals of log₂ expression levels and corrected IMF content, with FDR < 0.05 set as the threshold for statistical significance. To avoid bias from small-sample inter-group comparison, we integrated population‑level correlation results from all 79 pigs with our ATAC‑seq data. We found 108 of the 294 group-shared differential ACR-linked genes were significantly correlated to IMF content. A heatmap of these genes demonstrated that their expression levels varied with IMF content across the 79 Laiwu pigs (Fig. 5D). Specifically, 40 genes were upregulated and 40 genes were downregulated in regions with high chromatin openness, while 18 genes were downregulated and 10 genes were upregulated in regions with low chromatin openness (Fig. 5A). This differential expression pattern may be attributed to the regulatory effects of TFs or transcriptional repressors on gene expression.

**Fig. 6:**
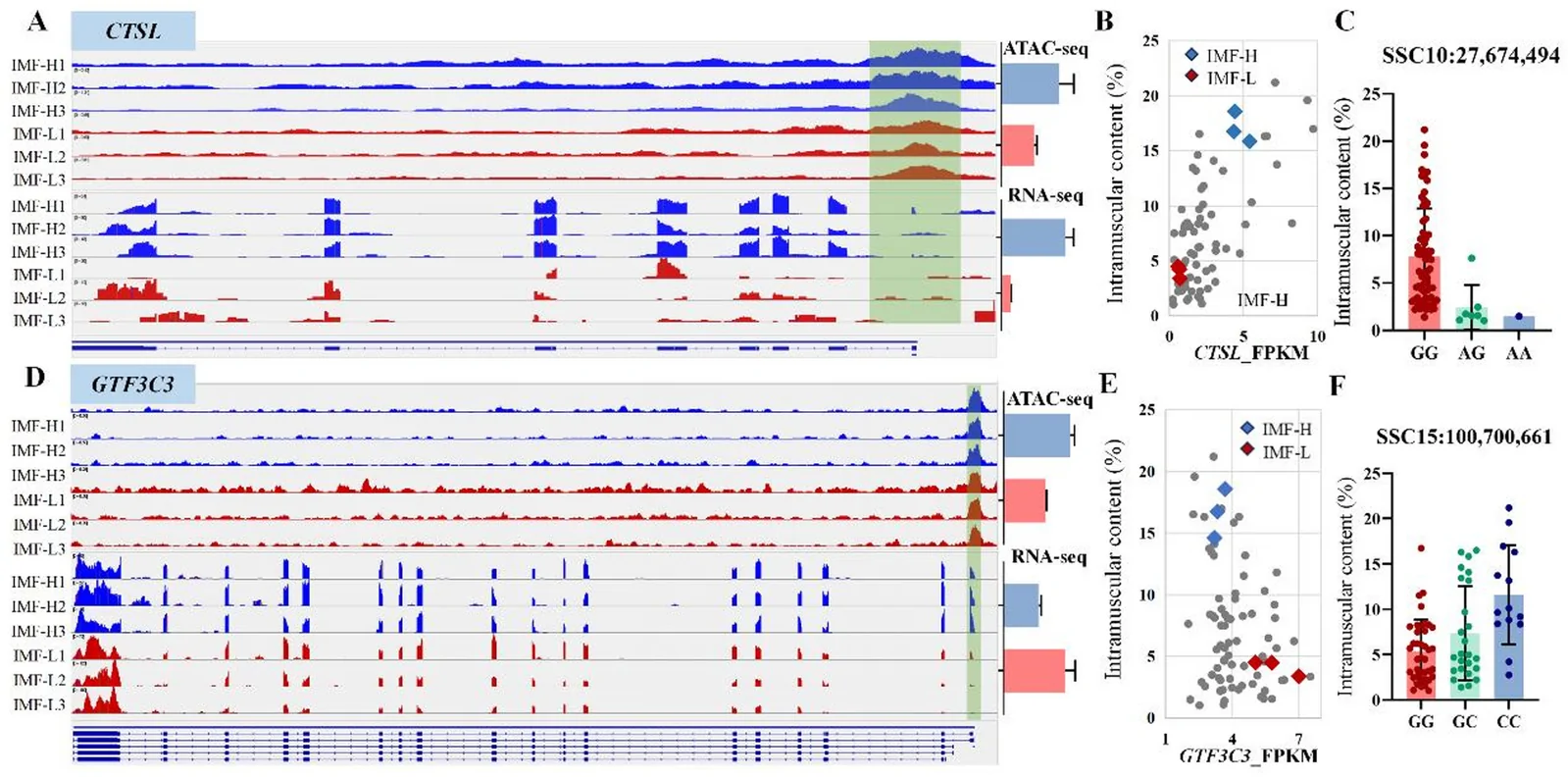
Identification of candidate genes regulating intramuscular fat (IMF) content through integrated ATAC-seq and RNA-seq analysis. **A** Peak profile and gene expression signal of *CTSL* showing accessible chromatin region (ACR) corresponding to *CTSL* expression. The green region denotes ACR corresponding to *CTSL*. **B** Scatter plot between *CTSL* expression and IMF content in 79 Laiwu pigs. FPKM: Fragments per kilobase of transcript per million mapped reads. **C** IMF content of different genotypes of a SNP (SSC10: 27,674,494) located in the ACR of *CTSL*. **D** Peak profile and gene expression signal of *GTF3C3* showing ACR corresponding to *GTF3C3* expression. The green region denotes ACR corresponding to *GTF3C3*. **E** Scatter Plot between *GTF3C3* expression and IMF content in 79 Laiwu pigs. **F** IMF content of different genotypes of a SNP (SSC15: 100,700,661) located in the ACR of *GTF3C3*

We then focused on the top 10 group-shared differential ACR-linked genes with the highest correlation with IMF content (Table 3). Five of these genes, including *IDH1* [39], *RORA* [40], *DGAT2* [41], *VCL* [42], and *CTSL*, have been reported to be involved in fat deposition. Notably, the ACR of *CTSL* was located at TSS of this gene. Both the chromatin accessibility and transcription level of *CTSL* were higher in IMF-H group than in IMF-L group (Fig. 6A). Consistent with this, our prior study identified a significant positive correlation between *CTSL* expression and IMF content (coefficient = 0.48, FDR = 2.07E-04, Fig. 6B). These findings suggest that elevated chromatin accessibility at the TSS of *CTSL* may drive the upregulation of its transcriptional level, ultimately contributing to the increase in IMF content. Within *CTSL* ACR, one SNP located at 27,674,494 on SSC10 was significantly associated with IMF content in the 79 Laiwu pigs (*p* = 1.13E-04) and explained 14.18% of the phenotypic variation (Table S6). About 90% of the individuals carried the GG genotype for this SNP, while only one individual had the AA genotype. Notably, the IMF content in individuals with the GG genotype (7.84 ± 0.59%) was significantly higher than that in those with the GA genotype (2.44 ± 0.82%, Fig. 6C).

**Table 3:** Top 10 group-shared differential accessible chromatin region-linked genes with the highest correlation coefficients with intramuscular fat (IMF) content

Gene Name	Gene ID	Peak ID	Peak log_2_FC	Peak annotation	Coefficient	FDR
*IDH1*	ENSSSCG00000023264	15:11334178–111,334,523	0.78	Distal Intergenic	0.58	5.24E-06
*RORA*	ENSSSCG00000004576	1:111208159–111,208,863 1:111317344–111,318,149	-0.91 -0.64	Distal Intergenic Distal Intergenic	-0.57	6.95E-06
*SNRPD2*	ENSSSCG00000033500	6:52031995–52,032,308	-0.91	Exon	0.54	2.56E-05
*KCMF1*	ENSSSCG00000008242	3:59656404–59,656,630	-0.64	Promoter (2-3 kb)	-0.54	2.66E-05
*DGAT2*	ENSSSCG00000034102	9:10060502–10,060,924	-0.91	Intron	0.51	7.27E-05
*SERPINH1*	ENSSSCG00000039468	9:9770984–9,771,321	-0.64	Distal Intergenic	0.50	8.63E-05
*MAP3K20*	ENSSSCG00000015960	15:79187760–79,188,210	-0.91	Intron	-0.50	8.63E-05
*VCL*	ENSSSCG00000010313	14:76750221–76,750,700	-0.64	Intron	-0.50	9.82E-05
*PTPN4*	ENSSSCG00000015743	15:31525977–31,526,465	-0.91	Distal Intergenic	-0.48	1.79E-04
*CTSL*	ENSSSCG00000010948	10:27674288–27,674,870	-0.64	Promoter ( < = 1 kb)	0.48	2.07E-04

Peak log_2_FC: Log_2_(fold change) of the group-shared differential peaks

Coefficient: Pearson correlation coefficient between correct IMF content and log₂-transformed gene expression

*FDR* False discovery rate, calculated using the Benjamini–Hochberg method

Additionally, the most significantly ACR spanned the TSS of the *GTF3C3* genes. The protein encoded by this gene is a component of TFIIIC2 complex, which binds to the promoters of small nuclear and cytoplasmic RNA genes, thereby facilitating the recruit of RNA polymerase III. For *GTF3C3*, chromatin accessibility was higher in the IMF-H group than in the IMF-L group, whereas the transcription level showed the opposite trend (Fig. 6D). Correspondingly, *GTF3C3* expression was significantly negatively correlated with IMF content (Coefficient = -0.46, FDR = 3.86E-04, Fig. 6E). These findings imply that *GTF3C3* may regulate IMF deposition through a mechanism where altered chromatin accessibility at its TSS modulates its own transcriptional level, thereby influencing the expression of downstream genes involved in IMF accumulation. Among the SNPs in the *GTF3C3* gene, a SNP at position 100,700,661 on SSC15 showed a significant association with IMF content in the 79 Laiwu pigs (*p* = 1.57E-04, Table S7). This SNP explained 13.63% of the phenotypic variation in IMF content. Supporting this association, individuals carrying the CC genotype exhibited a significantly higher IMF content (11.60 ± 1.40%) compared to those with the GG genotype (5.72 ± 0.63%, Fig. 6F).

### Validation of *CTSL* and *GTF3C3* gene expression and genotypes

qRT-PCR results revealed that the expression trends of *CTSL* and *GTF3C3* were consistent with RNA-seq data. Strong correlations were observed between the two detection methods, with correlation coefficients of 0.97 for *CTSL* and 0.96 for *GTF3C3*, corresponding to coefficients of determination *R*^2^ of 0.9385 and 0.9277 respectively (Fig. S2A). These results confirmed the reliability of gene expression profiles obtained from RNA-seq. Meanwhile, Sanger sequencing targeting the SNP sites chr10:27674494 of *CTSL* and chr15:100700661 of *GTF3C3* presented fully matched genotypes compared with whole-genome resequencing data (Fig. S2B).

## Discussion

Among the various meat quality traits, IMF content is of paramount importance as an economic trait. Epigenetic regulation has been increasingly recognized to impact meat quality in numerous farm animals [43], however, limited studies have explored the role of chromatin remodeling in IMF deposition. This research gap is particularly evident in studies comparing individuals with high and low IMF content within breeds inherently characterized by high IMF levels, as opposed to cross-breed comparisons.

Skeletal muscle is a heterogeneous tissue composed of myofibers, intramuscular adipocytes, fibroblasts, endothelial cells, and immune cells. Despite the substantially higher IMF content in Laiwu pigs compared to both commercial lean-type breeds and most other indigenous breeds [25, 44], IMF still constitutes a small fraction of the tested muscle tissue. Many studies have found that the development of IMF and skeletal muscle is not an independent process but rather a coordinated event involving intricate crosstalk between muscle cells and adipocytes [45, 46]. Against this background, we performed ATAC-seq on LT muscle samples collected from Laiwu pigs with extreme IMF phenotypes. Given that our ATAC-seq was performed on LT muscle tissue, the detected ACRs mainly reflect the landscape of myofibers, while also capturing regulatory signals from diverse cell types including intramuscular adipocytes in the muscle microenvironment. These data enable us to identify two categories of functional genes: one category may include regulatory genes in muscle cells that indirectly influence IMF content by modulating the muscle microenvironment or secreting signaling molecules, while the other may consist of genes in intramuscular adipocytes that may directly govern lipid biosynthesis, triglyceride storage, and adipocyte maturation.

In the IMF-H and IMF-L groups, we identified more than 40,000 peaks per group, respectively. The number of peaks obtained in our study was consistent with the findings of Yue et al.’s study [47], who identified 20,000–60,000 peaks in skeletal muscle during embryonic development in pigs. For comparison, a previous study in cattle reported 10,023 and 11,360 peaks in the muscle genomes of adult individuals and embryos, respectively [48]. Among the peaks of the IMF-H group, motif enrichment analysis revealed that the binding sites of JunB, Fra1, Atf3 and Fra2 were ranked among the top 10 most significantly enriched TF binding motifs. Functional evidence for these TFs in adipocyte biology have been well documented: reduced JunB expression in adipocytes expands the fraction of cells with robust thermogenic capacity, thereby conferring resistance to diet-induced obesity and insulin resistance [49, 50]; Fra2, which is expressed in the late stages of adipogenesis, binds to the promoters of *CIDEA*, *NOTCH1*, *ARAF*, and *MYLK* to regulate their expression and subsequent lipid metabolic processes [51]; the Fra-2/AP-1 complex modulates adipocyte differentiation and survival via the regulation of *PPARγ* and hypoxia signaling, while elevated Fra-1 expression induces severe lipodystrophy [52, 53]; and Atf3, a well-recognized therapeutic target for obesity and related metabolic disorders, exerts a negative regulatory effect on adipocyte deposition [54–56]. By contrast, motif enrichment analysis of the IMF-L group peaks indicated that the binding motifs of CTCF, Mef2c, Mef2d, and BORIS were significantly enriched. Notably, these TFs are widely recognized as core regulators essential for skeletal muscle development and myogenic differentiation [57–60]. Collectively, these results highlight a clear functional divergence between the two groups: the IMF-H group is characterized by the enrichment of adipocyte- and lipid metabolism-related TFs, whereas the IMF-L group is dominated by muscle development-associated TFs, which strongly correlate with their respective phenotypic traits of high and low IMF content.

To mitigate the impact of such intercellular heterogeneity of LT tissue on our results, we adopted a relatively loose screening strategy, using the FC > 1.5 to identify differentially ACRs between the high- and low-IMF groups, while prioritizing ACRs consistently present within each group. Notably, this employment of an FC > 1.5 threshold as a lenient screening criterion is consistent with previous ATAC-seq studies for differential peak selection [61]. The loose threshold effectively reduces the risk of false-negative results caused by heterogeneous cell compositions masking subtle but biologically relevant changes in chromatin accessibility. Meanwhile, focusing on differential peaks consistent among replicates in each group could ensure that the identified regions reflect consistent regulatory patterns associated with IMF phenotypes, rather than random fluctuations from individual-specific variations or technical noise.

For the integration analysis of ATAC-seq and RNA-seq data, most prior studies have prioritized the identification of overlapping genes between ACR-linked genes and RNA-seq-derived differentially expressed genes (DEGs) [18–20]. Different from this conventional strategy, we integrated ACR-associated genes with genes showing significant expression correlation with IMF content in 79 individuals, rather than DEGs identified between IMF-high and IMF-low groups. This modification was mainly determined by actual data characteristics. The sample size used for pairwise DEG comparison was relatively small, so many functional genes with slight expression changes would be easily excluded under strict screening thresholds. In addition, skeletal muscle is a heterogeneous tissue containing multiple cell types. Such cellular composition differences among individuals would produce more obvious interference on reliable DEG identification when the sample number is limited. In comparison, using population-wide phenotype correlation analysis can effectively avoid the above limitations. It can steadily capture genes with continuous expression changes along with IMF content variation, and is more suitable for heterogeneous tissue samples. Anchored on these IMF-correlated genes, our approach prioritized regulatory loci directly contributing to IMF content in Laiwu pigs and facilitated the construction of a more robust genotype-phenotype regulatory network linking chromatin accessibility dynamics to the transcriptional modulation of IMF-related genes.

Following the integrative analysis, we identified 108 ACR-linked genes that were significantly correlated with IMF content. Notably, the ACR of *CTSL*, a gene that exhibited a significantly positive correlation with IMF content, was located at TSS of this gene. Cathepsin L (CTSL/CATL) is a lysosomal protease; its expression and activity are impaired in the adipose tissue (AT) of obese rodents and humans, indicating AT lysosomal dysfunction [62, 63]. Elevated levels of *CTSL* have also been reported in obese and diabetic patients, with this upregulation exerting regulatory effects on adipogenesis and glucose tolerance [64]. In pigs, *CTSL* has been demonstrated to play a key role in meat qualitative traits through post-mortem proteolysis, as well as in quantitative production traits including average daily gain, lean meat, and backfat thickness [65, 66]. A known SNP in *CTSL* (AJ315771:g.143 C > T), located approximately 3,000 bp upstream of the TSS, has been shown to impact qualitative and quantitative traits in pigs [65]. However, in the present study, we detected a novel SNP (SSC10:27,674,494) within the ACR of *CTSL* that was associated with IMF content in Laiwu pigs, with its specific regulatory mechanism warranting further investigation.

Within the most significantly differential group-shared ACR, we identified the TF gene *GTF3C3*, which was significantly negatively correlated with IMF content. Accumulating evidence has linked *GTF3C3* to obesity: in preadipocytes isolated from obese humans with type 2 diabetes, *GTF3C3* expression exhibited a negative correlation with its methylation level [67]; meanwhile, in a porcine model for human obesity, *GTF3C3* exhibited higher connectivity in the obese vs. lean network and was differential expressed between lean and obese groups [68]. Consistent with these findings, gene-based association analysis in the pig biobank database (https://pigbiobank.farmgtex.org/) revealed that *GTF3C3* was significantly associated with lean cuts percentage (*p* = 1.14E-07) and with backfat thickness (*p* = 3.00E-05)—two traits closely related to systemic fat deposition—further supporting its critical role in adipose metabolism.

Collectively, our integrative multi‑omics analysis identified a set of candidate genes underlying IMF deposition in Laiwu pigs, among which *CTSL* and *GTF3C3* are two promising key candidates. In future studies, we will further verify the regulatory roles of *CTSL* and *GTF3C3* in adipogenesis and lipid metabolism using in vitro cellular models and in vivo functional experiments. We will also focus on identifying the key transcription factors that bind to the open chromatin regions of these two genes, and perform transcription factor footprinting and regulatory network analyses to characterize their transcriptional activity. Moreover, the novel molecular markers detected in this study will be validated in expanded populations and applied to marker‑assisted breeding for improving pork quality. These efforts will help establish a complete regulatory network governing IMF deposition and provide robust molecular targets for pig genetic improvement.

## Conclusion

In this study, we identified regions with differential chromatin accessibility associated with IMF content using ATAC-seq. The analysis was performed on six Laiwu pigs with extreme IMF phenotypes, selected from a population of 79 individuals. Compared with the IMF-L group, the IMF-H group exhibited 5,573 upregulated differential peaks and 3,806 downregulated differential peaks of chromatin accessibility, among which 525 differential peaks were shared within each group. By integrating ATAC-seq and RNA-seq data, we identified 108 group-shared differential ACR-linked genes that were significantly correlated with IMF content. Furthermore, we characterized several genes with promoter accessibility changes and expression levels significantly correlated with IMF content, including *CTSL* and *GTF3C3*, which may be involved in regulating IMF deposition. Collectively, our findings provide a valuable theoretical basis for subsequent investigations into the chromatin accessibility—an epigenetic regulatory process—and transcriptional regulatory mechanisms underlying IMF deposition in Laiwu pigs.

## Supplementary Information

Supplementary Material 1: Fig. S1. Top 10 signiﬁcantly enriched transcription factor binding motifs in high intramuscular fat content (A) and low intramuscular fat content groups (B). (C) Enrichment analysis of TF binding motifs from the top 10 significantly enriched motifs of the two groups.

Supplementary Material 2: Fig. S2. Validation of *CTSL* and *GTF3C3* gene expression by qRT-PCR and genotypes by Sanger sequencing. (A) Linear regression analysis of expression levels between RNA-seq and qRT-PCR data. The X-axis represents the relative expression detected by qRT-PCR, and the Y-axis indicates the FPKM values from RNA-seq. (B) Genotypes of the target SNPs confirmed by Sanger sequencing.

Supplementary Material 3: Table S1: Distribution of the accessible chromatin regions (ACRs) in high intramuscular fat (IMF) content (IMF-H) group and low IMF content (IMF-L) group. Table S2: Differential accessible chromatin peaks between high intramuscular fat (IMF-H) and low intramuscular fat (IMF-L) groups. Table S3: Differential accessible chromatin peaks among six individuals and strictly group-specific peaks between high intramuscular fat (IMF-H) and low intramuscular fat (IMF-L) groups. Table S4: Significantly enriched pathways (*p* < 0.05) from group-shared differential accessible chromatin-linked genes. Table S5: Significantly enriched Gene Ontology (GO) terms (*p* < 0.05) from group-shared differential accessible chromatin-linked genes. Table S6: Association analysis between SNPs in the accessible chromatin region (ACR) of the *CTSL* gene and intramuscular fat (IMF) content in 79 Laiwu pigs. Table S7: Association analysis between SNPs in the accessible chromatin region (ACR) of the *GTF3C3* gene and intramuscular fat (IMF) content in 79 Laiwu pigs

## Data Availability

All raw sequencing data have been deposited in the Genome Sequence Archive in National Genomics Data Center, China National Center for Bioinformation/Beijing Institute of Genomics, Chinese Academy of Sciences (GSA: CRA036706, submitted on 7 January 2026) that are publicly accessible at https://ngdc.cncb.ac.cn/gsa.
